# Antihyperlipidaemic and hepatoprotective activities of acidic and enzymatic hydrolysis exopolysaccharides from *Pleurotus eryngii* SI-04

**DOI:** 10.1186/s12906-017-1892-z

**Published:** 2017-08-14

**Authors:** Chen Zhang, Juan Li, Jing Wang, Xingling Song, Jianjun Zhang, Shang Wu, Chunlong Hu, Zhiyuan Gong, Le Jia

**Affiliations:** 1 Institute of Agricultural Resources and Environment, Shandong Academy of Agricultural Science, Key Laboratory of Wastes Matrix Utilization, Ministry of Agriculture, Jinan, 250100 China; 2 College of Life Science, Shandong Agricultural University, Taian, 271018 China; 3 Chinese Academy of Agricultural Sciences, Beijing, 100081 China; 4 The Central Hospital of Taian, Taian, 271000 China; 5 College of Food Science, Shandong Agricultural University, Taian, 271018 China; 6 College of Forestry, Shandong Agricultural University, Taian, 271018 China

**Keywords:** Antihyperlipidaemic, Hepatoprotective, Antioxidant, Acidic and enzymatic hydrolysis exopolysaccharides, *Pleurotus eryngii* SI-04

## Abstract

**Background:**

Hyperlipidaemia is the major risk factor contributing to the development and progression of atherosclerosis, fatty liver and cerebrovascular disease. *Pleurotus eryngii* (*P. eryngii*) is rich in biologically active components, especially polysaccharides that exhibit various biological activities, including reducing blood lipids. In the present study, three novel polysaccharide types, including exopolysaccharides (EPS), enzymatic EPS (EEPS) and acidic EPS (AEPS) were isolated, and the hypolipidaemic and hepatoprotective effects were investigated to better understand possible hypolipidaemic mechanisms and their hepatoprotective effects.

**Methods:**

The EPS was hydrolysed by snailase (dissolved in 1% acetic acid, pH = 6) and H_2_SO_4_ (1 M) to obtain EEPS and AEPS, respectively. The in vitro antioxidant activities were measured by investigating the reducing power and the scavenging effects on radicals of hydroxyl, 1,1-diphenyl-2-picrylhydrazyl (DPPH) and superoxide anion. The hyperlipidaemic mice were induced by perfusing a high-fat emulsion. In addition to the hepatic histopathology, the following biochemical analyses were performed to investigate the antioxidative effects, including the activities of alkaline phosphatase (ALP), alanine aminotransferase (ALT), aspartate aminotransferase (AST), glutathione peroxidase (GSH-Px), superoxide dismutase (SOD) and catalase (CAT). Triacylglycerol (TG), total cholesterol (TC), high-density lipoprotein cholesterol (HDL-C), low-density lipoprotein cholesterol (LDL-C), malondialdehyde (MDA) and lipid peroxidation (LPO) levels were also measured in serum and liver homogenate.

**Results:**

Supplementation of EPS, EEPS and AEPS could significantly improve blood lipid levels (TC, TG, HDL-C, and LDL-C), hepatic lipid levels (TC and TG), hepatic enzyme activities (ALP, ALT, and AST) and antioxidant status (GSH-Px, SOD, T-AOC, MDA, and LPO). In addition, histopathological observations indicated that these polysaccharides had potential effects in attenuating hepatocyte damage.

**Conclusion:**

These results demonstrated that both EPS and its hydrolysates EEPS and AEPS might effectively reduce serum lipid levels and protect against high-fat diet-induced hyperlipidaemia, indicating that they could be used as functional foods and natural hepatoprotectants.

## Background

High-fat diets provide excess energy intake and disrupt lipid metabolism, resulting in fat accumulation in many tissues, especially serum and liver [1]. Hyperlipidaemia, always caused by high-fat diets, is mainly characterized by increased levels of total cholesterol (TC), triglyceride (TG) and low-density lipoprotein cholesterol (LDL-C), along with a decrease in high-density lipoprotein cholesterol (HDL-C). These changes are the major risk factors contributing to the development and progression of atherosclerosis, fatty liver and cerebrovascular disease [2–5]. Many researchers have focused on the roles of oxidative damage and lipid peroxidation in the pathomechanism of hyperlipidaemia [6]. Oxidative stress, always caused by superfluous reactive oxygen species (ROS), is an early event in the evolution of hyperlipidaemia. ROS, including hydroxyl (HO·), DPPH· and superoxide (O^−^_2_·) radicals, are potentially toxic to various biological molecules, resulting in oxidative damage that can accelerate the pathogenic progress of hyperlipidaemia and its complications [6–8]. Under hyperlipidaemic conditions, enzymatic and non-enzymatic antioxidative defence systems such as superoxide dismutase (SOD), catalase (CAT) and glutathione peroxidase (GSH-Px) are altered, leading to ROS-mediated damage [9]. Scientists have suggested that the appropriate support for enhancing the antioxidant supply in subjects with hyperlipidaemia can attenuate the course of the disease. Maladjusted lipid synthesis and lipid clearance also play roles in causing hyperlipidaemia, and methods to reduce blood lipid levels could be effective in treating this disease [5]. Thus, effects on the antioxidant and hypolipidaemic properties of some bioactive compounds are particularly promising for improving human health [10].

Recently, the treatment of hyperlipidaemia has been involved in diet control, exercise and pharmaceutical therapy. Since synthetic lipid-lowering drugs, including statins and fibrates, usually have side effects and contraindications with long-term use, the application of natural hypolipidaemic drugs seems to be urgent to prevent and treat hyperlipidaemia and its complications [11]. *Pleurotus eryngii* (*P. eryngii*), one type of common edible fungus in China, is rich in biologically active components, including polysaccharides, peptide, sterols and dietary fibre [12]. As the most potent mushroom-derived substances, polysaccharides exhibit various biological activities, including antioxidant, anti-aging, antivirus and anti-lipid peroxidation properties. [13]. Furthermore, modified polysaccharides have received more attention due to their superior physicochemical properties, including good water-solubility, high stability, and non-toxicity [14]. Previous studies have shown that crude polysaccharides from the fruiting body of *P. eryngii* have potential effects in reducing blood lipids [15]. However, the hypolipidaemic effects of exopolysaccharides and their chemically modified forms have not been evaluated. In the present study, three kinds of novel polysaccharides – exopolysaccharides (EPS), enzymatic EPS (EEPS) and acidic EPS (AEPS) – were isolated, and their hypolipidaemic and hepatoprotective effects were investigated. EPS, EEPS and AEPS possessed hypolipidaemic and antioxidant activities, indicating that the polysaccharides could be developed as valuable functional foods/drugs for clinical hypolipidaemic and hepatoprotective treatments.

## Methods

### Strain and chemicals

The *P. eryngii* SI-04 strain was provided by the Fungi Institute of the Academy of Agricultural Sciences (Tai’an, China). The diagnostic kits for analysing SOD activities, GSH-Px activities, CAT activities, total antioxidant capacity (T-AOC) activities, lipid peroxidation (LPO) contents and malondialdehyde (MDA) contents were purchased from the Nanjing Jiancheng Bioengineering Institute (Nanjing, China). The standard monosaccharide samples, including rhamnose (Rha), ribose (Rib), arabinose (Ara), xylose (Xyl), glucose (Glc), mannose (Man) and galactose (Gal) were provided by the Merck Company (Darmstadt, Germany) and Sigma Chemical Company (St. Louis, USA). Other reagents and chemicals used in the present work were analytical reagent grade and were supplied by local chemical suppliers.

### Preparation of EPS

The liquid fermentation of *P. eryngii* SI-04 was processed using the method from our present work [16]. The EPS of *P. eryngii* SI-04 was obtained by referencing the method of Ma et al. (2015) with slight modifications. After centrifugation (3000 rpm, 15 min), the supernatant fermentation broth was mixed with 3 volumes of 95% ethanol (*v*/v), stirred thoroughly and stored at 4 °C for 24 h. The precipitate was deproteinized with Sevag reagent (chloroform/*n*-butanol, 5:1, *v*/v) and lyophilized by vacuum freeze-drying (Labconco, USA) to obtain EPS. The EPS was weighed, and the yield was 3.81 g/L.

### Enzymatic and acidic hydrolysis of EPS

The enzymatic hydrolysis of EPS was processed according to the methods of Yang et al. [17] and Li et al. [18] with some modifications. The polysaccharide sample (0.5 g) and snailase (0.1 g) were dissolved in 100 mL of 1% acetic acid at pH 6 and 37 °C for 4 h. After quick pre-freezing, the enzymatic hydrolysis exopolysaccharides (EEPS) were lyophilized for further analyses.

The acidic hydrolysis of EPS was processed according to the method of Ma et al. [19] with slight modifications. Briefly, EPS (0.5 g) was dissolved in 10 mL of 1 M H_2_SO_4_ solution, and the reaction was processed in a boiling water bath for 8 h. After centrifugation (6000 rpm, 10 min) and neutralization, the supernatant was concentrated and lyophilized to obtain acidic exopolysaccharides (AEPS).

### Monosaccharide composition analysis

The monosaccharide compositions of EPS, EEPS and AEPS were calculated using gas chromatography (GC-2010, Shimadzu, Japan) equipped with a flame ionization detector (FID) and an Rtx-1 capillary column (30 m × 0.25 mm × 0.25 μm). The samples and standard monosaccharides were pre-processed using our previous method [16]. The initial oven temperature of the column was maintained at 190 °C for 20 min and increased gradually to 200 °C at a rate of 3 °C /min. Nitrogen was used as the carrier gas at 0.8 mL/min of cavity flow and 19.8 mL/min of total flow. The samples (1.0 μL) were injected in the split model (1:20) at 260 °C. The monosaccharide content was expressed as the following formula:

1 $$ Monosaccharide content\ \left(\%\right)=\frac{A}{B}\times \frac{V}{M}\times C $$

where A and B were the peak areas of sample and standard monosaccharides, V was the sample constant volume (mL), M was the sample quality (g), and C was the monosaccharide concentration of the mixed standard (mg/mL).

### Antioxidant effects in vitro

The reducing power was assayed according to our previous work [16].

The scavenging capability on hydroxyl radicals was evaluated using the method of Koksal et al. [20] with few modifications. The reaction mixture, including 1 mL of phenanthroline (7.5 mM), 1 mL of ferrous sulphate (0.75 mM), 5 mL of phosphate buffer (pH 7.4), 1 mL of sample (0–1000 mg/L) and 1 mL of hydrogen peroxide (3%, *v*/v) was shaken sufficiently and incubated at 37 °C for 30 min. The absorbance was measured at 560 nm using distilled water as a blank, and the scavenging rate was calculated using the following formula:

2 $$ Scavenging rate\ \left(\%\right)=\frac{A-B}{B}\times 100 $$

where A was the absorbance of distilled water, and B was the absorbance of samples.

The scavenging capability on DPPH radicals was measured using the methods of Brand-Williams et al. [21] and Kong et al. [22] with some modifications. The reaction mixture, containing 2 mL of ethanol (95%, *w*/*v*), 0.1 mL of DPPH (l M) and 2 mL of sample (0–1000 mg/L), was incubated at room temperature and placed in the dark for 30 min. The absorbance of the solution was determined at 517 nm. The scavenging rate was evaluated using the following formula:

3 $$ Scavenging rate\ \left(\%\right)=\left(1-\frac{A}{B}\right)\times 100 $$

where A was the absorbance of the tested sample, and B was the absorbance of the blank.

The scavenging capability of superoxide anion radicals was measured using the method of Stewar and Beewley [23] with slight modification. Briefly, 1.0 mL of sample (0–1000 mg/L) was added to the mixture containing phosphate-buffered saline (0.5 mL, 0.2 M, pH 7.8), riboflavin (0.3 mL, 10 mM) and methionine (0.25 mL, 13 mM), and the reaction was incubated at 25 °C for 30 min. The absorbance of the solution was determined at 560 nm, and the scavenging rate was calculated using the following formula:

4 $$ Scavenging rate\ \left(\%\right)=\left(1-\frac{A}{B}\right)\times 100 $$

where A was the absorbance of polysaccharide samples, and B was the absorbance of the blank.

### Experimental design

#### Preparation of high-fat emulsion

The high-fat emulsion was prepared using the method of Zhao, Huang and Yuan [11] with sight modifications. Briefly, the oil phase, including 25 g lard oil, 10 g cholesterol, 1 g methylthiouracil and 25 mL of Tween-80, was heated to the melting point on a magnetic stirring apparatus (Guohua Instrument Ltd. Co. Changzhou, China). Simultaneously, the water phase contained 30 mL distilled water, 20 mL propylene glycol and 2 g sodium deoxycholate. Subsequently, the water and oil phases were mixed thoroughly before animal administration.

#### Design of the animal experiment

Seventy-two Kunming strain mice (20 ± 2 g, male), purchased from Taibang Biological Products Ltd. Co. (Tai’an, China) were housed in polycarbonate cages and freely accessed food and water ad libitum at constant conditions of 22 ± 1 °C and constant humidity (50 ± 5%) under a 12-h light-dark cycle.

After adapting to the environment for 7 d, all mice were weighed and randomly distributed into nine groups (eight mice per group). In the hyperlipidaemia group (HL), mice were perfused with high-fat emulsion alternated with distilled water. Mice in the simvastatin group (ST) were perfused with high-fat emulsion alternated with simvastatin (200 mg/kg body weight). In the other six treatment groups (L-EPS, H-EPS, L-EEPS, H-EEPS, L-AEPS and H-AEPS), mice were perfused with high-fat emulsion alternated with EPS, EEPS and AEPS at 400 and 800 mg/kg body weight. Mice in the normal control group (NC) were given distilled water daily, and the entire experiment lasted 28 days. All the experiments were submitted to and approved by the ethics committee of the Shandong Agricultural University.

After overnight fasting, all mice were weighed and sacrificed under anaesthesia. The serum was obtained by centrifugation (10,000 rpm, 10 min) from blood in the retrobulbar vein. The livers were excised, weighed and homogenized (1:9, *w*/*v*, in normal saline and ethyl alcohol). After centrifugation (5000 rpm, 20 min, 4 °C), the supernatants were collected and stored at 0 °C for further biochemical analysis.

#### Biochemical and histopathological assays

Alkaline phosphatase (ALP), alanine aminotransferase (ALT) and aspartate aminotransferase (AST) activities and TG, TC, HDL-C and LDL-C levels in serum were measured using an automatic biochemical analyser (ACE, USA). GSH-Px, SOD and CAT activities in serum/liver homogenate and the MDA, LPO, TC and TG contents in liver homogenate were analysed using commercial kits according to the instructions.

The liver tissue staining method followed a previously published study [16].

#### Acute toxicity assay

The acute toxicity test in mice was performed on the basis of the reported method [24] with some modifications. The mice were randomly divided into four groups, including one control group and three dose groups (eight mice per group). The mice in dose groups received intragastric administration with EPS, EEPS and AEPS at 5000 mg/kg body weight, while the control group received isometric saline solutions. All mice had free access to food and water ad libitum for 10 days under regular observation for any mortality or behavioural changes, including irritation, restlessness, respiratory distress, abnormal locomotion and catalepsy.

### Statistical analysis

All data are presented as the means ± standard deviations (SD) of three independent experiments. Significant differences among groups were determined by one-way ANOVA (SPSS 16.0 software package, USA). *P* < 0.05 was considered statistically significant.

## Results

### Monosaccharide composition analysis

**Fig. 1:**
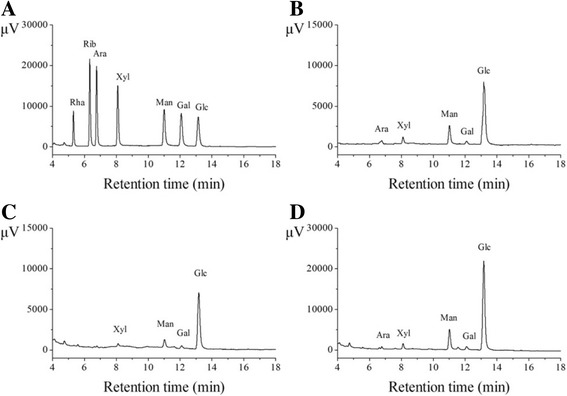
Gas chromatographs of (**a**) standard monosaccharides, (**b**) EPS, (**c**) EEPS, and (**d**) AEPS

The monosaccharide compositions of EPS, EEPS and AEPS were identified according to the retention times and chromatograph peaks using monosaccharide guide samples (Fig. 1a). The EPS consisted of five different monosaccharides, including Ara, Xyl, Man, Gal and Glc, in mass percentages of 3.42%, 4.76%, 17.03%, 4.46% and 70.32%, with a molar ratio of 1:1.5:4.3:1.1:17.7 (Fig. 1b). The EEPS consisted of three different monosaccharides, including Man, Gal and Glc, in mass percentages of 12.85%, 4.93% and 82.22%, with a molar ratio of 1:4:1.9:31.2 (Fig. 1c). The AEPS consisted of five monosaccharides, including Ara, Xyl, Man, Gal and Glc, at mass percentages of 1.36%, 2.86%, 14.50%, 2.99% and 78.03%, with a molar ratio of 1:2.1:15.7:1.8:47.7 (Fig. 1d).

### Antioxidant effects in vitro

**Fig. 2:**
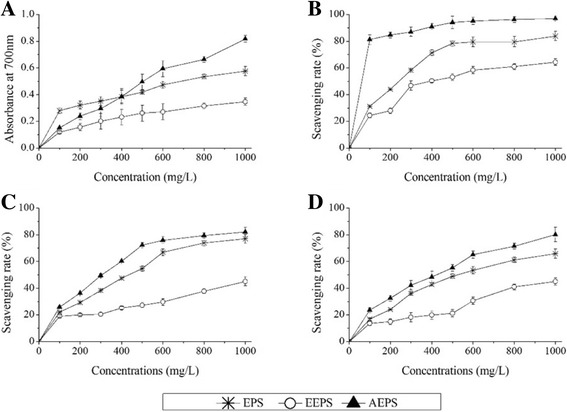
Antioxidant activities of EPS, EEPS and AEPS in vitro. (**a**) reducing power, (**b**) hydroxyl radicals, (**c**) DPPH radicals, and (**d**) superoxide anion radicals

In this study, the in vitro antioxidant capacities of EPS, EEPS and AEPS were estimated with reducing power using the HO·, DPPH· and O^−^_2_· systems. As shown in Fig. 2a, the polysaccharides could cause the reduction of the Fe^3+^/K_3_Fe(CN)_6_ complex to Fe^2+^, as monitored by measurement of the enhanced formation of Perl’s Prussian blue at 700 nm [25]. In the broad range of 100–1000 mg/L, the reducing power of AEPS ranged from 0.152 ± 0.016 to 0.817 ± 0.025, which was much higher than those of EPS and EEPS, indicating that AEPS had the potential to be explored as a stronger antioxidant. Furthermore, AEPS showed significant scavenging effects against HO· at 100 mg/L, with the scavenging rate reaching 81.14 ± 3.71%, which was even higher than that of EEPS (64.37 ± 2.53%) at 1000 mg/L (Fig. 2b). Similarly, EPS, EEPS and AEPS had obvious scavenging effects against DPPH radicals, and the scavenging activities increased with increasing concentration. The scavenging rate of AEPS reached 82.17 ± 3.51% at 1000 mg/L, which was 36.92 ± 1.27% and 7.22 ± 0.61% higher than those of EPS and AEPS, respectively (Fig. 2c). For the scavenging activities on O^−^_2_·, as exhibited in Fig. 2d, AEPS showed superior scavenging activity of O^−^_2_· compared with EPS and EEPS in a dose-dependent manner. At 1000 mg/L, the scavenging rates of AEPS, EPS and EEPS reached 80.21 ± 2.24%, 65.91 ± 3.14% and 45.23 ± 1.75%, respectively. In conclusion, the superoxide radical-scavenging activity of AEPS was stronger than those of EPS and EEPS.

### Effects on body weight and the hepatosomatic index (HI)

The effects of EPS, EEPS and AEPS on body weight and the hepatosomatic index of hyperlipidaemic mice are shown in Table 1. Mice in the HL group exhibited a significant increase in body weight compared with the NC group (*P <* 0.001) on both the 10th and 20th days. Polysaccharide administrations at different doses could cause significant decreases in body weight in hyperlipidaemic mice (*P* < 0.05 or *P* < 0.001) on the 20th day. Simultaneously, a significant increase in HI (liver weight/body weight (g/100 g) could be observed in the HL group after treatment with high-fat emulsion (*P* < 0.001). However, this uptrend could be mitigated by pre-treatment with EPS, EEPS and AEPS at 400 and 800 mg/kg body weight, indicating that they could suppress hepatic steatosis to some extent. Simvastatin had similar effects as the samples.

**Table 1:** Effects of EPS, EEPS and AEPS on body weights and HI of high-fat mice

Groups	Body weight (g)			HI (%)
	The 1st day	The 10th day	The 20th day	The 20th day
NC	29.13 ± 0.21	32.19 ± 1.69	34.18 ± 1.02	3.92 ± 0.19
HL	29.29 ± 0.15	39.29 ± 1.53^###^	43.24 ± 1.96^###^	5.63 ± 0.26^###^
ST	29.5 ± 0.19	35.29 ± 1.01^**^	37.27 ± 2.36^**^	4.32 ± 0.15^***^
L-EPS	28.97 ± 0.11	36.3 ± 1.9	38.96 ± 1.55^**^	4.95 ± 0.12^*^
H-EPS	29.24 ± 0.23	36.68 ± 1.56	38.19 ± 1.57^**^	4.62 ± 0.21^***^
L-EEPS	29.23 ± 0.32	36.92 ± 1.88	40.21 ± 1.96	5.43 ± 0.16
H-EEPS	28.96 ± 0.17	36.81 ± 1.77	38.99 ± 1.20^**^	5.17 ± 0.17
L-AEPS	29.04 ± 0.07	36.27 ± 1.39	39.02 ± 0.92^**^	4.78 ± 0.23^**^
H-AEPS	29.22 ± 0.12	35.01 ± 2.21^**^	37.21 ± 1.30^***^	4.26 ± 0.11^***^

The values were reported as the mean ± SD of eight mice per group

^###^Significant difference compare to NC, *P* < 0.001

*Significant difference compare to MC, *P* < 0.01

**Significant difference compare to MC, *P* < 0.05

***Significant difference compare to MC, *P* < 0.001

### Biochemical assays in blood serum

The serum lipid levels, which usually contain TC, TG, HDL-C and LDL-C clinically, are shown in Table 2. After perfusion of a high-fat emulsion, the serum TG, TC and LDL-C levels of mice in the HL groups were significantly (*P* < 0.001 or *P* < 0.05) increased, while the HDL-C (*P* < 0.05) levels were markedly decreased compared with the NC group, indicating successful model construction in hyperlipidaemic mice. After 20-day gavage administration, these pathologic changes were markedly mitigated by pre-treatment with EPS, EEPS and AEPS at two different dosages (*P* < 0.01, *P* < 0.05 or *P* < 0.001), respectively. As shown in Table 2, after treatment with AEPS at a dose of 800 mg/kg body weight (H-AEPS group), the TG, TC and LDL-C levels reached 1.65 ± 0.21, 2.29 ± 0.06, and 0.61 ± 0.02 mM, lower than those of EPS (1.80 ± 0.15,2.89 ± 0.13 and 0.62 ± 0.03 mM) and EEPS (1.99 ± 0.16, 3.41 ± 0.21 and 0.81 ± 0.05 mM), while the HDL-C levels reached 1.92 ± 0.14 mM, higher than those of EPS (1.82 ± 0.05 mM) and EEPS (1.65 ± 0.12 mM) for the same dose groups, respectively. Treatment with simvastatin in the ST groups also showed potential effects against increased TG, TC and LDL-C levels and decreased HDL-C levels.

**Table 2:** Effects of EPS, EEPS and AEPS on TG, TC, HDL-C and LDL-C levels in serum

	TG	TC	HDL-C	LDL-C
NC	2.14 ± 0.11	2.16 ± 0.09	1.95 ± 0.12	0.42 ± 0.03
HL	2.81 ± 0.19^###^	5.81 ± 0.96^###^	1.65 ± 0.07^##^	1.09 ± 0.05^###^
ST	2.09 ± 0.21^***^	3.67 ± 0.28^***^	2.01 ± 0.06^***#^	0.56 ± 0.04^***^
L-EPS	1.82 ± 0.17^***^	3.15 ± 0.36^***^	1.72 ± 0.14	0.83 ± 0.07^**^
H-EPS	1.80 ± 0.15^***^	2.89 ± 0.13^***^	1.82 ± 0.05^**^	0.62 ± 0.03^***^
L-EEPS	2.06 ± 0.13^***^	3.75 ± 0.34^***^	1.68 ± 0.13	0.91 ± 0.04^*^
H-EEPS	1.99 ± 0.16^***^	3.41 ± 0.21^***^	1.65 ± 0.12	0.81 ± 0.05^**^
L-AEPS	1.72 ± 0.14^***^	2.49 ± 0.11^***^	1.79 ± 0.06^**^	0.74 ± 0.07^***^
H-AEPS	1.65 ± 0.21^***^	2.29 ± 0.06^***^	1.92 ± 0.14^**^	0.61 ± 0.02^***^

The values were reported as the mean ± SD of eight mice per group

^##^Significant difference compare to NC, *P* < 0.05

^###^Significant difference compare to NC, *P* < 0.001

*Significant difference compare to MC, *P* < 0.01

**Significant difference compare to MC, *P* < 0.05

***Significant difference compare to MC, *P* < 0.001

**Fig. 3:**
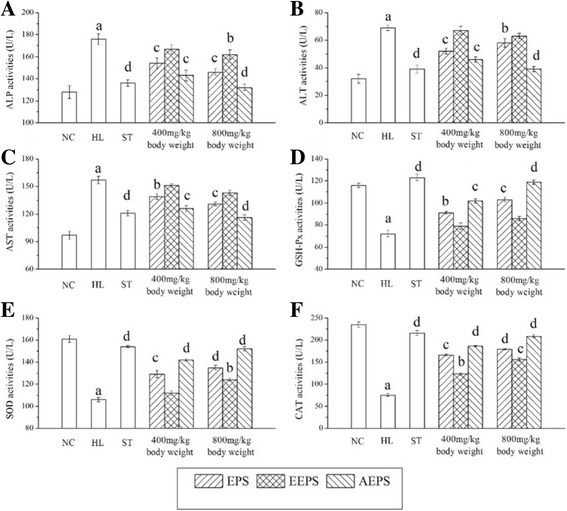
Effects of EPS, EEPS and AEPS on activities of (**a**) ALP, (**b**) ALT, (**C**) AST, (**d**) GSH-Px, (**e**) SOD and (**f**) CAT in serum. The values were reported as the mean ± SD of eight mice per group. *a* Significant 306 difference compare to NC, *P* < 0.001. *b* Significant difference compare to MC, *P* < 0.01. *c* Significant difference compare to MC, *P* < 0.05. *d* Significant difference compare to MC, *P* < 0.001

The serum enzyme activities, including ALP, ALT, AST, GSH-Px, SOD and CAT, were assayed, and the results are displayed in Fig. 3. The ALP, ALT and AST activities in the HL group were significantly higher (*P* < 0.001), while the GSH-Px, SOD and CAT activities were markedly lower than those in the NC group (*P* < 0.001), indicating that liver damage was induced by oxidative stress. Interestingly, as shown in Fig. 3, EPS, EEPS and AEPS had potential effects in decreasing high levels of ALP, ALT and AST (*P* < 0.01, *P* < 0.05 or *P* < 0.001) while increasing the low activity levels of GSH-Px, SOD and CAT at the tested dosage, respectively.

### Biochemical assays in liver

**Fig. 4:**
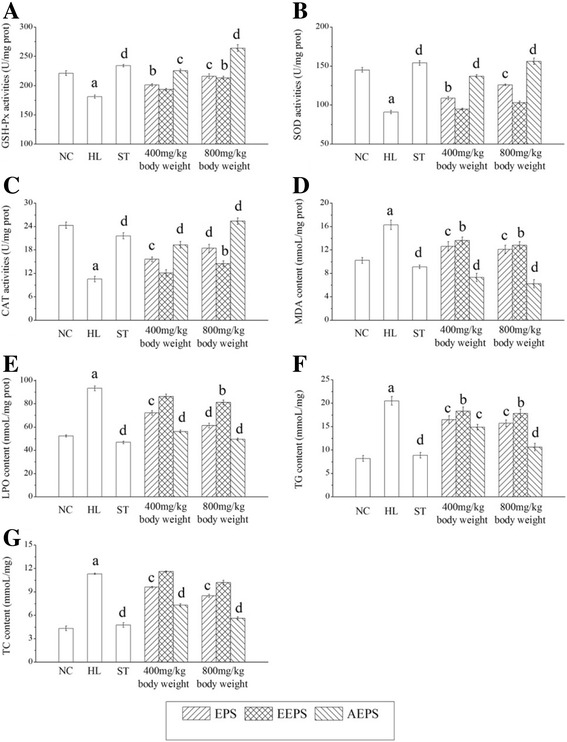
Effects of EPS, EEPS and AEPS on the hepatic activities of (**a**) GSH-Px, (**b**) SOD and (**c**) CAT, as well as contents of (**d**) MDA, (**e**) LPO, (**f**) TG and (**g**) TC. The values were reported as the mean ± SD of eight mice per group. The values were reported as the mean ± SD of eight mice per group. *a* Significant difference compare to NC, *P* < 0.001. *b* Significant difference compare to MC, *P* < 0.01. *c* Significant difference compare to MC, *P* < 0.05

The effects of EPS, EEPS and AEPS on hepatic enzyme activities, lipid peroxidation and lipid contents are shown in Fig. 4. Obvious, significant decreases in GSH-Px, SOD and CAT activity levels and significant increases in MDA, LPO, TG and TC contents were observed in the HL group compared with the NC group, indicating that serious oxidative stress and accumulated lipids occurred in the liver (*P* < 0.001). Interestingly, these pathological changes could be alleviated by treatment with the three tested polysaccharides (EPS, AEPS and EEPS). After gavage with AEPS at the high dosage, hepatic GSH-Px and SOD activities were even higher than in the NC group. The effects on MDA and LPO contents were contrary to those for the enzyme activities. Regarding lipid contents, after 20 days of intragastric administration with AEPS, the hepatic TG levels in the L-AEPS and H-AEPS groups decreased by 27.32% and 48.29%, while the TC levels reduced by 35.41% and 50.44%, respectively.

### Liver histopathological observation

**Fig. 5:**
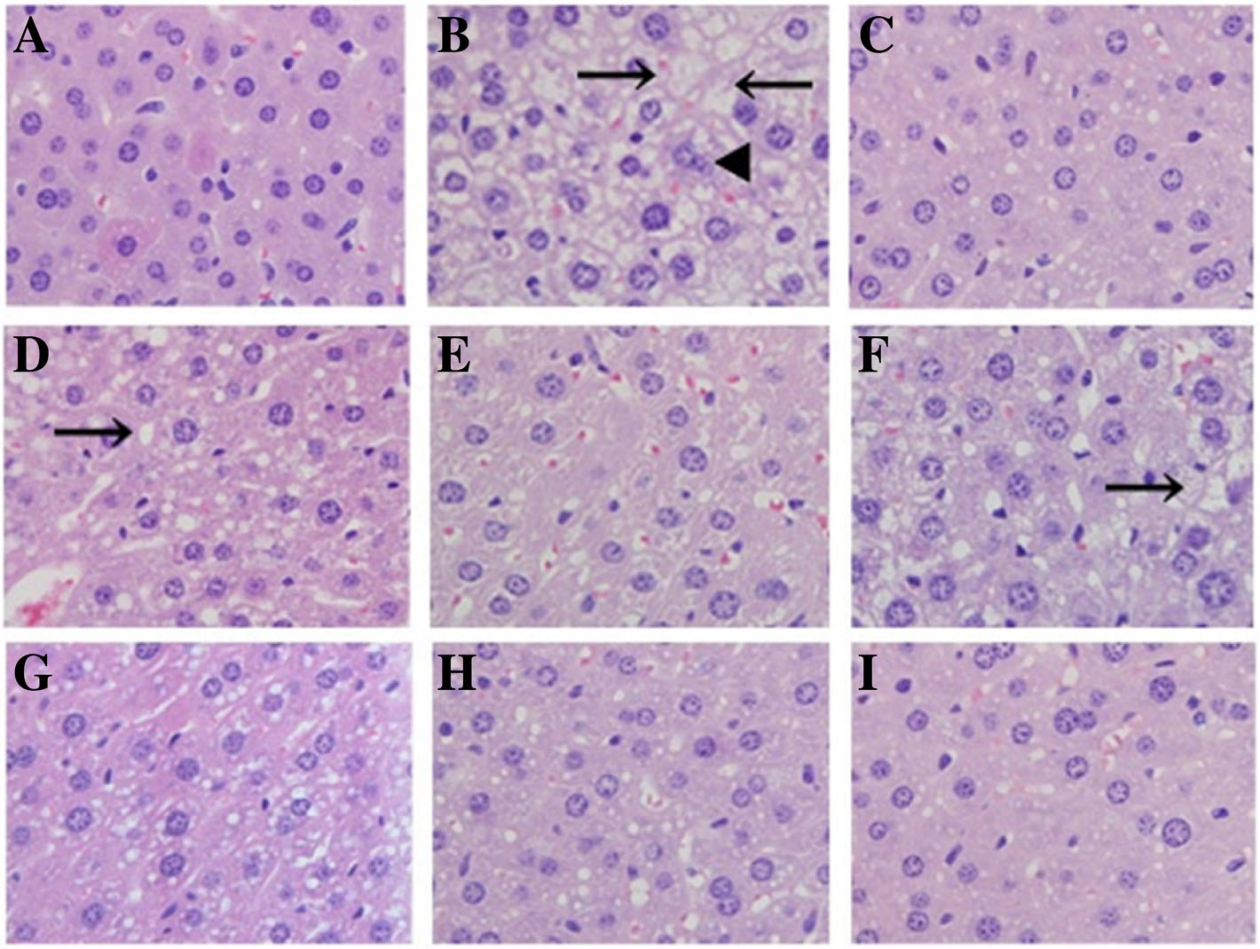
Effects of EPS, EEPS and AEPS on hepatic cells in the hyperglycemia mice (hematoxylin-eosin staining, 400×). (**a**) NC group, (**b**) HL group, (**c**) ST group, (**d**) L-EPS, (**e**) H-EPS, (**f**) L-EEPS, (**g**) H-EEPS, (**h**) L-AEPS and (**i**) H-AEPS (*n* = 8). *Arrows* show fat granule in cell, *triangles* indicate condensation or disappearance of cellular nucleus

Hepatocyte morphological changes were observed by optical microscope and are shown in Fig. 5. The liver was badly affected by the infusion with high-fat emulsion, showing extreme swelling, diffuse hepatic steatosis, inflammatory changes, fat droplets, vesicular degeneration and disappearance of nuclei compared with normal and well-arranged cellular morphologies, including abundant cytoplasm, distinct nuclei and well-defined cell borders without any fat degeneration (Fig. 5a and b). Interestingly, morphological liver structures in the dosage groups were significantly recovered. After EPS, EEPS and AEPS treatment, fat vacuoles were markedly reduced, and hepatocyte degeneration obviously decreased. The hepatocyte morphology and arrangement in the H-AEPS group were almost normal compared with NC mice, indicating that AEPS had evident inhibitory effects against high-fat emulsion-induced morphologic changes and liver steatosis. Moreover, the hepatocyte morphology of mice in the ST group indicated that simvastatin had the same effects.

### Acute toxicity assays

In acute toxicity assays, mice treated with EPS, EEPS and AEPS did not exhibit clinical signs of toxicity immediately or during the post-treatment period at a dosage of 5000 mg/kg body weight compared with the control group, indicating that these three polysaccharides were essentially non-toxic substances.

## Discussion

The scientific literature has indicated that hyperlipidaemia plays a very important role in the developmental progress of non-alcoholic fatty liver disease, atherosclerosis and cardiovascular disease [26, 27]. Several serum parameters, including elevated TC, TG and LDL-C levels and reduced HDL-C levels, are often considered indicators of hyperlipidaemia and are involved in increased risk of clinical diseases [28], consistent with the present results (Table 2). As the main carrier of cholesterol, excess LDL-C can be deposited in blood vessel walls, directly inducing the formation of atherosclerosis. High levels of HDL-C had protective effects because HDL-C can transport cholesterol from peripheral tissues to the liver through the “reverse cholesterol transport” pathway for catabolism [1, 29, 30]. In addition, TG levels play key roles in the regulation of lipoprotein interactions in maintaining normal lipid metabolism and have also been proposed as major determinants of cholesterol esterification, transfer and HDL remodelling in human plasma [31]. However, the variation trends in lipid levels are significantly mitigated by treatment with these three polysaccharides (EPS, EEPS and AEPS), indicating that the polysaccharides extracted from the fermentation broth of *P. eryngii* SI-04 showed positive antihyperlipidaemic effects on restoring high-fat emulsion-induced lipid metabolic disturbance. Ren et al. [32] demonstrated that these polysaccharides might be combined with lipids in lipid metabolism, accelerating transport and excretion of serum lipids.

Previous literature has reported that oxidative stress, usually induced by ROS and motivationally accelerating the development of endothelial damage and atherosclerosis – owing to its oxidative roles for the destruction of the nucleic acids, proteins and lipids of endothelial cell membranes – may be regarded as a possible mechanism to induce hyperlipidaemia [33, 34]. Lipid peroxidation could be a very sensitive biomarker for investigating the antioxidant effects, since lipid peroxidation could lead to hydroperoxide generation to toxic chemicals such as MDA. Excess MDA can oxygenate and modify LDL-C to form MDA-LDL-C, which can cause the degeneration and necrosis of endothelial cells, inflammatory reactions and disordered antioxidant systems [35, 36]. Experimentally, the major antioxidant enzymes, such as SOD, GSH-Px and CAT, were commonly used as biomarkers reflecting the production of free radicals and can prevent oxidative damage cooperatively at different sites during ROS metabolic pathways [37]. In the current study, serum GSH-Px, SOD and CAT activities decreased significantly (Fig. 2) after the perfusion of high-fat emulsion. The results were in accordance with those reported in previous articles [38, 39]. The significant and dose-dependent increases in these enzyme activities after treatment with AEPS indicated that AEPS had superior activity in the treatment of hyperlipidaemia.

In addition, excessively accumulated lipids in the liver can damage hepatic biomembranes, leading to an imbalance in oxidative phosphorylation and accelerating ROS formation. The imbalance of oxidation and reduction can cause lipid peroxidation and produce significant toxic intermediate products in the liver, resulting in hepatic necrosis and apoptosis [40]. Furthermore, oxidative stress can also produce an inflammatory reaction through cell injury, causing the infiltration of the liver parenchyma by inflammatory cells [11], in accordance with the results of the hepatocyte morphological assay (Fig. 5). The hepatocytes showed obvious diffuse hepatic steatosis and inflammatory changes in the HL group, and treatment of the samples alleviated these symptoms.

Moreover, it is well known that the biological activities of polysaccharides are always associated with their monosaccharide compositions [41]. The EPS consists of five monosaccharides, including Ara, Xyl, Man, Gal and Glc, in contrast to a previous conclusion for intracellular polysaccharides (IPS) from *P. eryngii* SI-04 [16]. Compared with the published literature, Chen et al. [10] demonstrated that the polysaccharides of the *P. eryngii* fruit body were mainly composed of Man, Glc and Gal. The difference in monosaccharide compositions may be related to the composition of the culture medium and the fermentation, extraction and purification conditions of polysaccharides [42]. Additionally, Wu et al. [14] demonstrated that the polysaccharides showed higher biological activities after hydrolysis with various glycosidases or acidic reagents. After enzymatic and acidic hydrolysis, the monosaccharide compositions and percentage compositions of EPS were altered. The results of in vitro antioxidant and antihyperlipidaemic assays indicated that AEPS with more abundant monosaccharide compositions than EEPS and higher Glc percentages than EPS performed better in these assays (Fig. 1).

## Conclusion

In summary, EPS and its two hydrolysates (EEPS and AEPS) were successfully obtained from *P. eryngii* SI-04. Their antihyperlipidaemic, antioxidant and hepatoprotective activities were also investigated. AEPS exhibited potential and impressive prevention effects on high-fat diet-induced hyperlipidaemia in mice that were similar to those of the prophylactic agent simvastatin, demonstrating that polysaccharides can be exploited as potential natural drugs and functional foods for the prevention and treatment of hyperlipidaemia.

## Abbreviations

AEPS: Acidic exopolysaccharides
ALP: Alkaline phosphatase
ALT: Alamine aminotransferase
Ara: Arabinose
AST: Aspertate aminotransferase
CAT: Catalase
DPPH: 1,1-diphenyl-2-picrylhydrazyl
EEPS: Enzymatic exopolysaccharides
EPS: Exopolysaccharides
Gal: Galactose
Glc: Glucose
GSH-Px: GSH peroxide
HDL-C: high-density lipoprotein cholesterol
HI: Hepatosomatic index
IPS: Intracellular polysaccharides
LDL-C: Low-density lipoprotein cholesterol
LPO: Lipid peroxidation
Man: Mannose
MDA: Malondialdehyde
Rha: Rhamnose
Rib: Ribose
ROS: Reactive oxygen species
SOD: Superoxide dismutase
T-AOC: Total antioxidant capacity
TC: Total cholesterol
TG: Triacylglycerols
Xyl: Xylose
